# Brazing Coupling Performance of Piezoelectric Waveguide Transducers for the Monitoring of High Temperature Components

**DOI:** 10.3390/s21010094

**Published:** 2020-12-25

**Authors:** Jiu Hong Jia, Ze Hou Wang, Dai Feng Yao, Shan-Tung Tu

**Affiliations:** Key Laboratory of Pressure Systems and Safety, Ministry of Education, East China University of Science and Technology, Shanghai 200237, China; wzh@mail.ecust.edu.cn (Z.H.W.); y30180587@mail.ecust.edu.cn (D.F.Y.); sttu@ecust.edu.cn (S.-T.T.)

**Keywords:** brazing coupling, piezoelectric waveguide transducers, high temperature, structural health monitoring

## Abstract

Piezoelectric waveguide transducers possess great potential for the online monitoring of high temperature critical components, in order to improve their operational safety. Due to the use of a waveguide bar, the sensory device is not susceptible to high temperature environments, which enables the long-term service of the piezoelectric transducers. However, the coupling between the waveguide bar and the high-temperature component has been proven to be the most important part of the monitoring system. In order to effectively transmit waves through the junction of the waveguide bar and the monitoring target, it is necessary to research a reliable coupling method to connect the waveguide transducers with the host structure. In the present research, the feasibility of brazing coupling for wave propagation through the junction was investigated through experiments. Piezoelectric waveguide transducers were welded using various kinds of brazing filler metals. The experimental results indicate that the coupling effects of the brazing welding depend on the filler metals. At the same time, some filler metals for the effective coupling of the transducer and the target monitoring component were identified. The brazing coupling method was verified that it can non-dispersively and effectively propagate waves into the host structure with much better reliability than the conventional dry coupling approach. Moreover, the high-temperature experimental results show that the brazing-coupled waveguide bar system can work reliably and stably in high temperatures at 300 °C for a long time. This work strives to pave a solid foundation for the application of piezoelectric waveguide transducers for the structural health monitoring of high temperature critical components.

## 1. Introduction

Pipes are the key components of nuclear power plants. Many serious accidents have been associated with the failure of pipes. A carbon steel pipeline of the second circuit of the Surry nuclear power plant in the United States was reported to have cracked, causing casualties in 1986 [1]. The wall thickness thinning of a high-pressure safety injection pipeline in Tihange nuclear power plant led to leakage in 1988 [2]. In 1991, two welding seams in the small pipes of the injection systems of Belleville nuclear power plant in France were found to have leaked [2]. In 1999, the excess discharge pipeline of the Number 2 machine in the Japanese Mihama nuclear power plant fractured [3]. In addition, the inlet nozzle of a regulator spray pipeline in Qinshan power plant leaked in 2013 [4]. The failures of the pipeline usually lead to the unexpected shutdown of plants, which may result in serious economic losses, and may even cause catastrophic accidents.

Non-destructive tests are normally carried out during plant shutdowns. The current non-destructive testing (NDT) techniques procedure, however, has several disadvantages, which may include the following: (1) the inspections have to be performed during shutdowns, with the possibility of prolonging the shutdown time and increasing production losses; (2) the insulating material wrapped around the pipes has to be removed and replaced for each manual measurement; and (3) the oxide scale formed on the measuring area should be cleaned, and scaffolding has to be built to inaccessible areas within the plant. Obviously, these drawbacks result in a considerable cost for the inspections. The financial consequences of prolonging shut-downs—which may be of the order of millions of dollars per day—have previously aroused a strong interest in the development of ultrasonic transducers that can operate at elevated temperatures above 285 °C for a long time.

For the safety of power plants, structural health monitoring (SHM) using ultrasonic technology has been investigated as a powerful way to quantitatively monitor, online, for cracks developing in this equipment, and the wall thinning rate of corrosion [4,5,6,7]. However, the lack of sensors that can sustain their function in such harsh environments for a long time considerably impedes the development of the extreme-environmental SHM systems, since the piezoelectric sensors that are widely used in the realm of SHM would lose their transduction capability beyond the Curie temperature. Even though some specially-tailored ultrasonic transducers may survive in these harsh environments, they still turn out to be vulnerable during long time service operations [8,9,10]. For instance, electromagnetic acoustic transducers and laser ultrasonic technology are outstanding non-destructive testing (NDT) approaches, but they cannot act as a permanently-installed online SHM system [11,12,13,14]. On the other hand, the waveguide bar systems have shown superb prowess in the monitoring of critical equipment in harsh environments. In such a system, the sensory device is mounted on the distal end of the waveguide bar with the high temperature components connected with the proximal end, so that the high temperature environments would not impose much influence on the sensory region [15,16,17].

In order to better transmit waves, a great deal of research has been carried out on the optimal design of waveguide bars. Heijinsdijk disclosed a coiled foil waveguide for the non-dispersive transmission of Lamb waves [18]. Cawley et al. employed an elongated strip as the waveguide bar in order to transmit certain shear modes of ultrasonic waves with advantageous non-dispersive characteristics [19]. The authors also investigated a design methodology of waveguide bars for the propagation of pure shear horizontal waves [20]. Kwon et al. put forward a tapering waveguide bar to survive the quasi SH0 wave and filtered some other shear horizontal waves [21]. Wang proposed a U-shaped waveguide bar to generate and receive the pure SH0 waves [22].

However, the coupling of the waveguide bar and the target specimen has not been investigated yet, despite its being crucial to the reliability and performance of the SHM and NDT system, i.e., the coupling quality will affect the signal energy and wave dispersion. Lawren advised the fusion welding of the metallic bar onto the test piece for high temperature monitoring [23], but Cegla proved by experiments that the welding will diffuse the waves [24]. Dry-coupling has been verified as a useful method for waveguide transducers [25], but the wave transmission efficiency cannot meet the needs for the thickness measurement of some important equipment [26]. In order to find a reliable coupling method to monitor critical equipment working in harsh environments, several brazing materials were tested by experiments in the present research. A certain brazing material that can couple the waveguide transducer at 300 °C was found. The coupling results were compared with those of dry coupling. The coupling performance was also verified through high temperature experiments.

## 2. Setup of the Experimental System for the Waveguide Transducer Coupling Performance Tests

### 2.1. Experimental Specimen

In order to monitor, online, the safety condition of pipes operating in high temperature (>200 °C) environments, the fundamental shear horizontal wave (SH0) is preferred. There are several beneficial aspects of the utilizing of the SH0 mode for SHM. An apparent beneficial aspect is the non-dispersive feature, which would reduce the signal processing and interpretation difficulties. Another benefit resides in the fact that the particle motion of the SH0 mode is parallel to the surfaces of the plate, and there is no out-of-plane particle displacement, so it is less affected by the presence of surrounding media. According to the research results in the current literature [20,22], a strip-bar of a large width-to-thickness ratio is the closest practical approximation to the anti-plane shear line, which can excite the SH0 mode wave. Therefore, the waveguide bar was designed in the form of a strip. The dispersion characteristics in infinite plates and in strip-bars are not identical. Strictly speaking, the term ‘fundamental shear horizontal wave’ doesn’t make sense in a strip-bar other than an infinite plate. Hence, the name ‘quasi-fundamental shear horizontal wave’ mode (shortened to SH0*) was chosen to indicate the ‘fundamental shear horizontal wave’ in a strip-bar.

In consideration of the high temperature equipment used in process industry, the material of the waveguide bars and specimens was selected as SUS 316L. The structural geometric parameters were set as 18 mm × 100 mm × 1 mm and 18 mm × 100 mm × 2 mm for the two waveguide bars.

**Figure 1:**
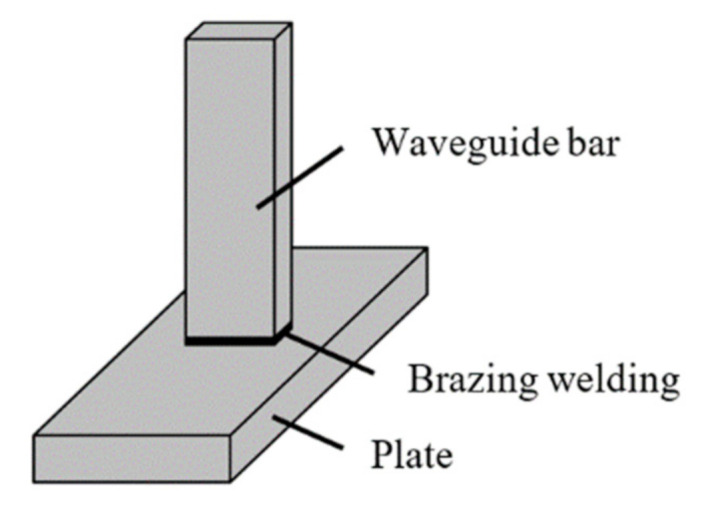
Schematic of the brazing specimen.

Each specimen comprised a waveguide strip and a plate, as shown in Figure 1. The plate structure had dimensions of 50 mm × 80 mm × 10 mm. The waveguide bars were brazed onto the plate.

**Figure 2:**
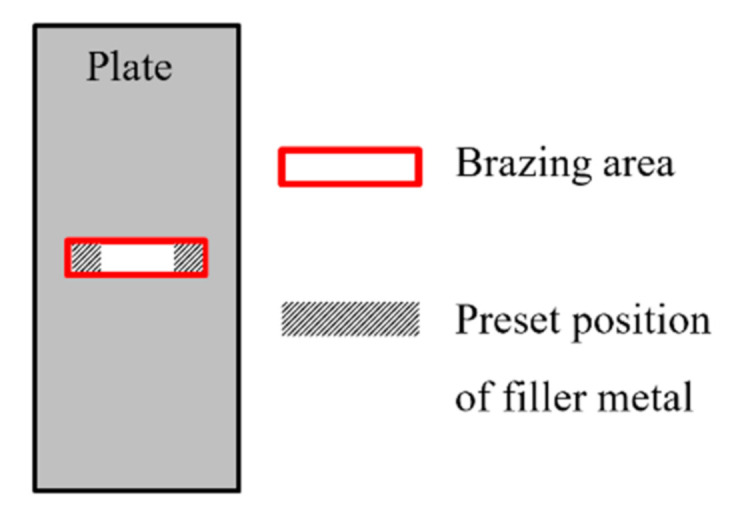
Schematic of the position of the brazing filler metals before brazing.

Brazing is a kind of welding method. The base metal will not be melted, while the brazing filler metal can sufficiently fill the junctions between the strip and the plate through wetting and capillary flow. The brazing method assembles the strip on the plate, so that the junction between the strip and the plate holds firm in order to propagate the waves. In the present research, three kinds of brazing filler metals that can potentially work at high temperatures were analyzed, as listed in Table 1. The brazing process took place as follows: first, a standard 0.1 mm-thick slice of brazing material was cut into a rectangular shape. The width of the brazing material slice was equal to the thickness of the waveguide bar, and the length of it was equal to one quarter of the width of the bar. Secondly, the filler metal was placed on two sides of the junction between the strip and the plate, as illustrated in Figure 2. In this way, less filler metal was consumed and the entire seam could be filled in well. Then, the brazing filler metals were heated up until they were completely melted. In order to avoid the oxidization of the base metals, brazing flux (QJ305) was painted onto the joints before the application of the heat. The heating temperatures were chosen according to the melting temperature of the filler metal, as presented in Table 1.

### 2.2. Experimental System

**Figure 3:**
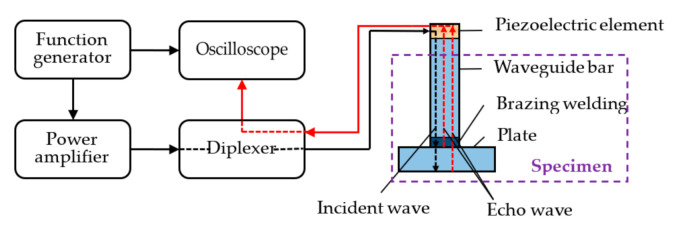
Schematic of the experimental setup.

The experimental system was set up as shown in Figure 3, and was comprised of a function generator (AFG 3021C, Tektronix, INC., Beaverton, OR, USA), a power amplifier (AG1006, T&C Power Conversion, INC., Rochester, NY, USA), a diplexer (Ritec, INC., Warwick, RI, USA), an oscilloscope (MDO3012, Tektronix, INC., Beaverton, OR, USA), a transducer (V154-RM, Olympus NDT, INC., Waltham, MA, USA), and a plate. The diplexer reduces the signal noises and controls the signals in order to send and receive waves on the same transducer orderly. A five-cycle pulse signal modulated by a Hanning window was generated by the function generator. Part of the generated signal was displayed in the oscilloscope, while the other part of the generated signal was enlarged by the signal amplifier in order to excite the transducer to generate shear horizontal waves on the top of the waveguide bar. The echo waves went through the diplexer and were recorded and displayed by the oscilloscope. The experiments were carried out using each waveguide strip specimen at several different central frequencies.

A total number of six specimens were fabricated using the aforementioned three brazing filler metals. The two waveguide bar dimensions with different thickness were tested for each of these three filler types. For the convenience of the description, these specimens were numbered, as shown in Table 2.

### 2.3. Effect of the Brazing Filler Metals in the Waveguide Bar

Signals at different frequencies were acquired using these specimens. All of the signals were acquired and compared. Some of the sensing signals demonstrated useful quality for SHM and NDT applications. The waves propagated through the joints efficiently between the waveguide bars and the plate, and were then reflected from the bottom of the plate. The echo waves could be clearly recorded by the transducer. However, it is apparent that the filler type imposed a great deal of influence on the wave coupling’s quality because the wave degrades so fast that there are no echoes from the plate bottom to be detected. This phenomenon represents substantial energy attenuation as waves propagated through the junction of the bar and the plate.

**Figure 4:**
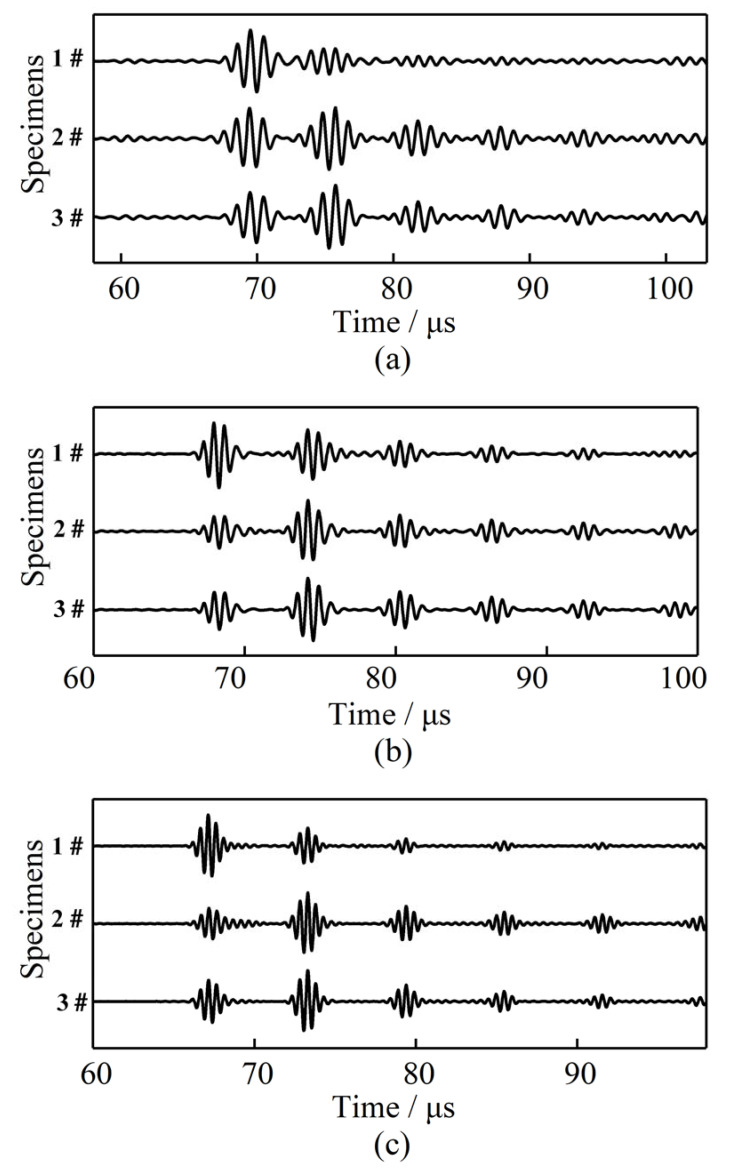
Waveforms in the specimens brazed with the waveguide bar of 1 mm thickness at different frequencies: (**a**) 1 MHz, (**b**) 1.5 MHz, and (**c**) 2 MHz.

The experiments were further carried out at three generally-applied frequencies in real engineering evaluations (1 MHz, 1.5 MHz and 2 MHz), and using three specimens with 1 mm thickness, with the representative waveforms shown in Figure 4. The wave packets from left to right were depicted as follows: the echo wave of the bar bottom, and the first, second, third, and fourth echo waves of the plate surface boundaries. The more echo waves of the plate identified, the more wave energy propagated through the brazing joints. More echo waves signify good coupling performance. Generally speaking, the first and the second echo waves could meet the requirements for NDT and SHM. However, more echo waves may significantly improve the accuracy of the detection. Let us take the thickness measurement of the plate as an example.

**Figure 5:**
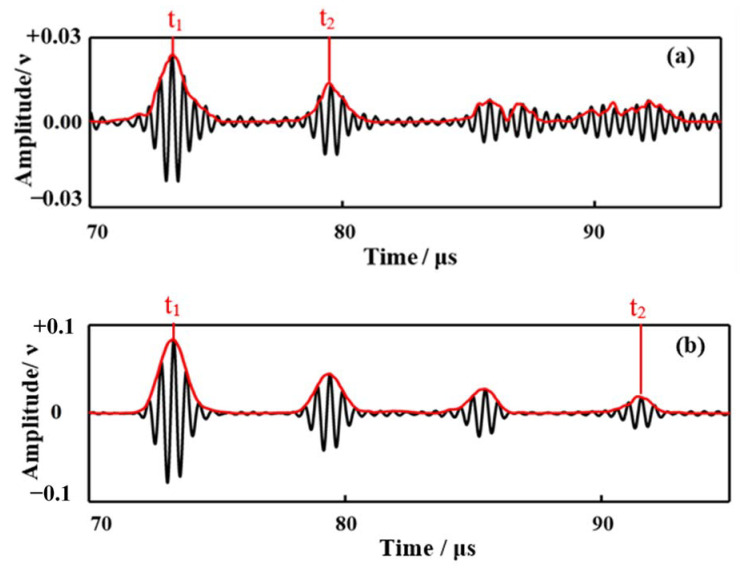
Waveforms with different degrading rates. (**a**) A fast degrading waveform (**b**) A slow degrading waveform.

According to the time of flights (TOF), the thickness of the plate *d* can be calculated by the equation:

(1) $$d=\frac{t_{n}-t_{1}}{2\left(n-1\right)}*v$$

where *v* is the wave velocity, $t_{1}$ is the time of the first echo wave of the plate bottom, $t_{n}$ is the time of the *n*th echo wave of the plate bottom, and *n* is the maximum number of wave packets that can be clearly measured. When *v* is known, the precision of the thickness calculation is really dependent on the accuracies of the TOF measurement, on the condition that the same signal processing method is used and the $\Delta t_{error}$ is assumed in each time extraction. Since the quality of the waveform degrades with the number of reflections, only the first two wave packets can be utilized in Figure 5a. In this case, the error of the thickness measurement is

(2) $$\Delta d=\frac{(t_{2}+\Delta t_{error})-\left(t_{1}-\Delta t_{error}\right)}{2}*v-\frac{t_{2}-t_{1}}{2}*v\;=\Delta t_{error}*v$$

However, there are four wave packets that can be utilized in Figure 5b. In this case, the error of the thickness measurement is

(3) $$\Delta d=\frac{(t_{2}+\Delta t_{error})-\left(t_{1}-\Delta t_{error}\right)}{6}*v-\frac{t_{2}-t_{1}}{6}*v\;=\frac{1}{3}*\Delta t_{error}*v$$

Therefore, the error in Equation (2) is one third of that in Equation (1). In other words, the more echo waves can be detected, the higher the accuracy of the measure. Based on this principle, there are some conclusions that can be drawn according to Figure 4. It was found that the waveforms of the echo signals propagating in 1# specimen were good, at 1.5 MHz and 2 MHz. The third echo of the plate bottom can be seen in Figure 4b, and the second echo wave can be clearly detected in Figure 4c. The waves degrade too quickly at 1 MHz, as shown in Figure 4a. The first echo of the plate bottom can be seen vaguely, but the second echo is mixed up with the noise signals. It cannot be utilized in NDT and SHM. The echo signals in the #2 and #3 specimens were good for all of the frequencies. The fourth echoes can be clearly detected. These phenomena may substantiate that the waves can effectively propagate through the welding joints with very little energy attenuation.

**Figure 6:**
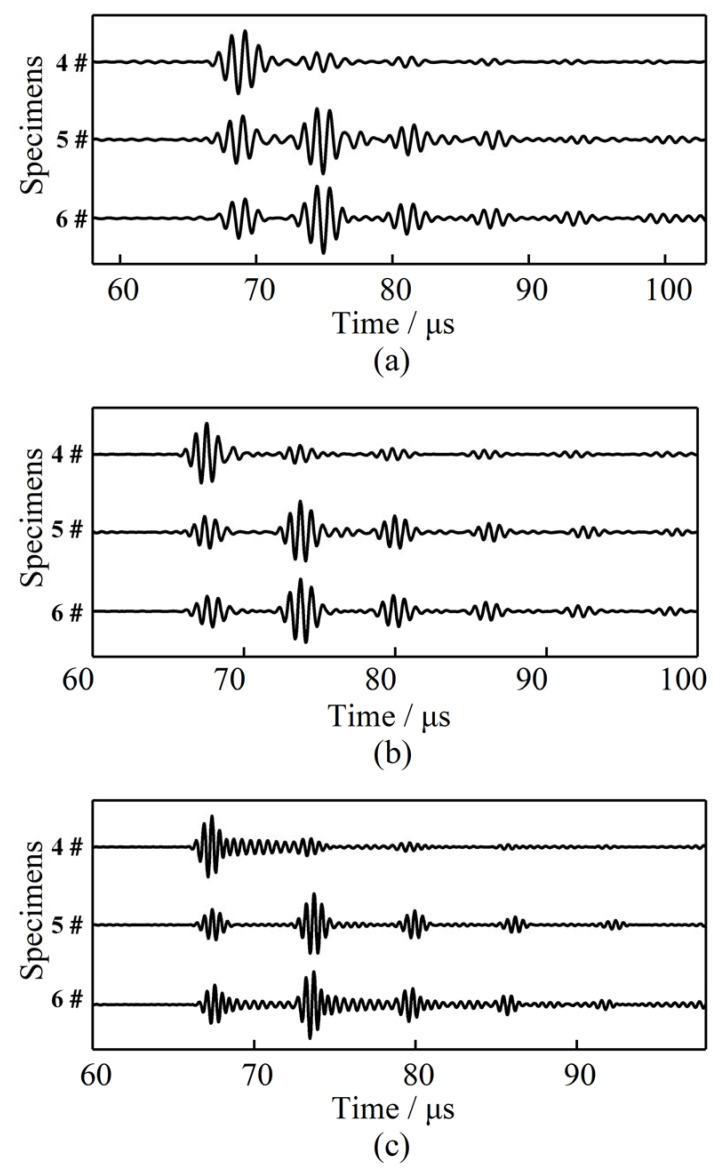
Waveforms in the specimens brazed with a waveguide bar of 2 mm thickness at different frequencies: (**a**) 1 MHz, (**b**) 1.5 MHz, and (**c**) 2 MHz.

In order to analyze the influence of the structural size on the coupling effect, experiments were also conducted using 2 mm-thick waveguide bars. The waveforms of the echo signals propagating in the specimens at three frequencies (1 MHz, 1.5 MHz, and 2 MHz) are presented in Figure 6. When the thickness of the waveguide bar was changed to 2 mm, the connecting area of the brazing joint became larger; on this condition, the signal changed slightly with the connecting area. It is easily noticed from Figure 6 that only the first echo wave packets of the plate bottom can be detected clearly when the #4 specimen is analyzed. The quality of the waveform degrades too quickly to be utilized in engineering. The #5 specimen and the #6 specimen can excite more wave packets. The third echo waves can all be detected.

### 2.4. Experimental Conclusions

According to the waveforms tested using the different specimens, it can be concluded that different filler metals would render different coupling effects. The brazing welding for the coupling is frequency-dependent when the filler metal isn’t a good fit. On the contrary, the brazing welds can couple the junction between the bar and the plate very well when the filler metal is suitable. The experimental results demonstrated that BAg45CuZn and BAg56CuZnSn could serve as good brazing filler metals in order to ensure outstanding coupling performance for wave propagating through the solid junctions when the base material is SUS 316L.

## 3. Comparison between Brazing Coupling and Dry Coupling

**Figure 7:**
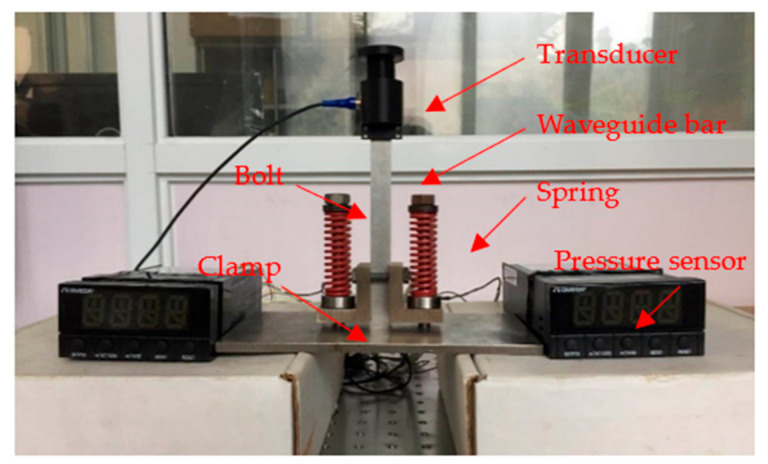
Installation of the waveguide bar using the dry coupling method.

In order to compare the performance of the brazing coupling and the dry coupling, a mechanical installation system was designed, as shown in Figure 7. The clamp was fixed onto the plate through bolts passing through the threaded holes in the plate. A specially-designed clamp pressed the waveguide bar onto the plate. The pressure sensor installed between the bolt and the clamp was used to measure the compressive force between them, so as to apply pressure evenly on both sides of the waveguide bar. During the process of mounting the bar, the pressing force was increased gradually until the echo signal reflected from the bottom of the plate was optimal. The waveguide bar mounted on the plate by dry coupling was called a dry coupling specimen, and that mounted by brazing was called the brazing coupling specimen.

**Figure 8:**
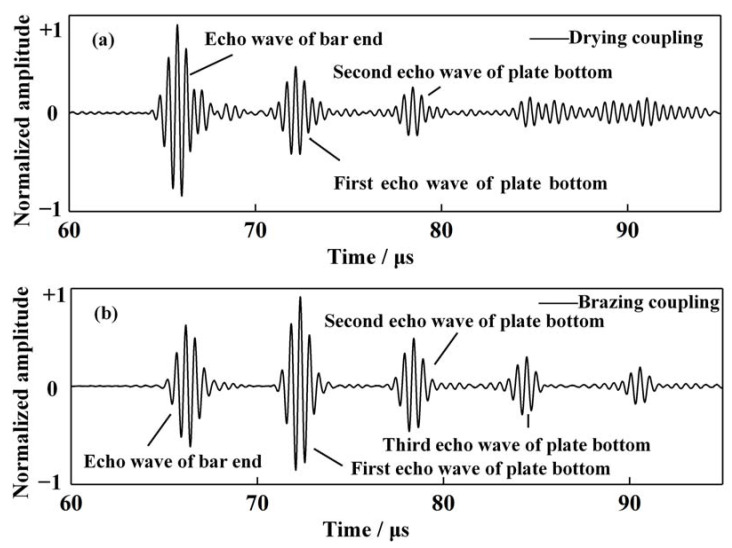
Waveforms comparison between the dry coupling specimen and the brazing coupling specimen. (**a**) Waveforms in the dry coupling specimen. (**b**) Waveforms in the brazing coupling specimen.

The other parameters in the current comparative test remained the same as those in the experimental system described in Section 2.2. The #2 brazing specimen was selected with the brazing material of BAg45CuZn. The waveguide bar dimensions were 18 mm × 100 mm × 1 mm. The waveforms of the brazing coupling system and the dry coupling system in the time domain are plotted in Figure 7. It is notable that both the dry coupling and the brazing coupling could work with good performance. However, the wave transmission efficiency of the brazing coupling proved to be much higher than the dry coupling, which can be inferred from the echo waves from the plate bottom. During the tests, the echo waves of the end of the waveguide bars in the dry coupling specimen and the brazing coupling specimen were adjusted to approximately equal amplitudes, as described by the echo waves of the bar end in Figure 8a,b.

In this manner, the amplitude of the echo waves from the bottom of the plate may serve as a good indicator of the coupling performance. If we define the transmission efficiency of coupling *η* as

(4) $$\eta =\;\frac{A_{pi}}{A_{b}}$$

where $A_{pi}$ is the *i*th echo wave of the plate bottom, *i* equals 1,2,3 or 4.

The transmission efficiency of each echo wave is listed in Table 3. The transmission efficiencies of the first and second echo waves from the plate bottom in the brazing coupling specimen were nearly three times as much as that in the dry coupling specimen. Furthermore, the fourth echo waves from the plate bottom in the brazing coupling specimen could be clearly observed. Nevertheless, the waves after the second wave packet in the dry coupling specimen were nearly mixed up with the background noise. In conclusion, brazing coupling demonstrated a more reliable and stronger capability to propagate waves than dry coupling.

## 4. Reliability of the Waveguide Bar Using Brazing Coupling

The conventional ultrasonic transducer and the waveguide bar system coupling were used to measure the same standard specimen at room temperature. The standard specimens are plates. Their thickness are 6.210, 9.840, and 20.104. The waveforms generated by the conventional ultrasonic transducer and the waveguide bar system were the same.

Their measurement errors for the two different methods are listed in Table 4. When the center frequency of the excitation signal was changed in the frequency range of 1–2.25 MHz, as is generally used in engineering, the measurement results remained approximately unchanged. The absolute errors of the measured values were less than 2%. These experiments demonstrated that the waveguide bar system using the brazing coupling could work reliably at different excitation frequencies.

## 5. Brazing Coupling Performance for High Temperature Applications

**Figure 9:**
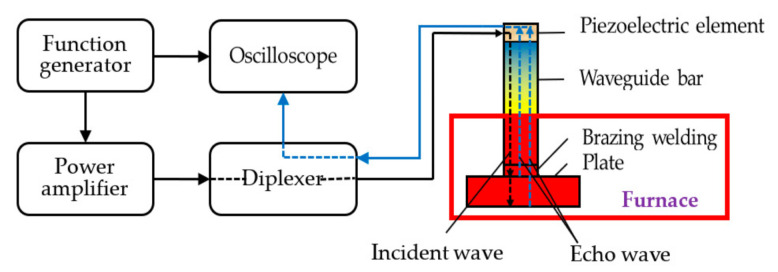
Schematic of the high temperature experimental system.

**Figure 10:**
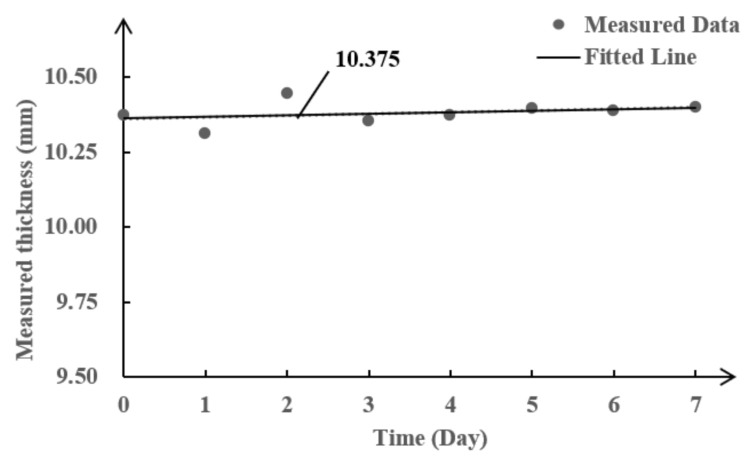
Long-term stability of the thickness measurement.

**Figure 11:**
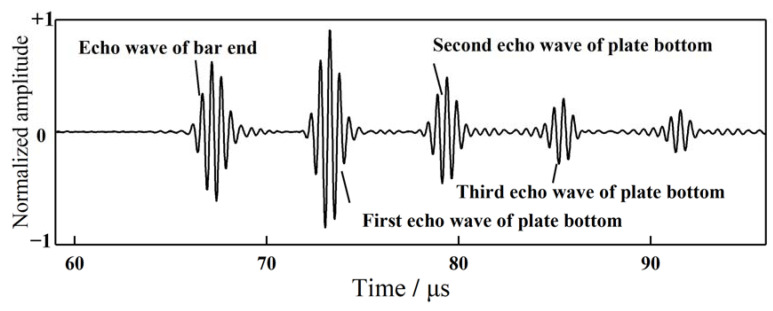
Waveforms of the brazing coupling specimen at 300 °C.

For the high temperature performance testing, the specimen and the proximal end of the waveguide bar were placed in a high temperature furnace, as presented in Figure 9. The distal end of the waveguide bar protruded from the small square window in the upper surface of the furnace. The piezoelectric sensory region resided outside the furnace. After the temperature arrived at the 300 °C, the environmental status was maintained for one week in order to investigate the long-term stability of the monitoring system. In the period of holding time, the signals remained stable; the thickness values were plotted in Figure 10. All of the waves were excited strongly in the pure SH mode. The waveforms are depicted in Figure 11. The thickness of the plate was evaluated as being 10.375 mm using the wave velocity calculated in reference [27]. The measurement error was 0.5%; that is to say, the brazing coupled waveguide bar system can be applied to measure the thickness of the plate reliably and stably at high temperatures.

## 6. Conclusions

In order to develop a good coupling method for piezoelectric waveguide transducers for the long term monitoring of high-temperature critical components, brazing coupling between the waveguide bar and the specimen was investigated. Various kinds of filler metals were selected according to their melting points and wave transmission characteristics. It was found that BAg45CuZn and BAg56CuZnSn could serve as good brazing filler metals for SUS 316L, in order to ensure outstanding coupling performance for wave propagating through the solid junction. Waveguide bars were brazed on a plate using the proposed filler metals. Experiments were carried out using these specimens at different exciting frequencies. The experimental results verified that the brazing coupling could non-dispersively and effectively propagate ultrasonic waves through the solid junctions between the waveguide bar and the specimen. Furthermore, it was demonstrated that the brazing coupling proved to be more reliable for the propagation of waves than the dry coupling. Moreover, the performance of the brazing-coupled ultrasonic waveguide transducers was experimentally investigated and demonstrated at 300 °C for a long term. The results substantiated that the brazing-coupled waveguide bar system possessed outstanding prowess for the reliable and stable measurement of the thickness of the plate in high-temperature environments.

## Figures and Tables

**Table 1:** Brazing filler metals and their melting temperatures.

Filler Metal	Melting Temperature(°C)
BAg40CdCuZn(Ni)	595~605
BAg45CuZn	665~745
BAg56CuZnSn	620~655

**Table 2:** Descriptions of the specimens.

Number	Filler Metal	Thickness of Waveguide Bar
1#	BAg40CdCuZn(Ni)	1 mm
2#	BAg45CuZn	
3#	BAg56CuZnSn	
4#	BAg40CdCuZn(Ni)	2 mm
5#	BAg45CuZn	
6#	BAg56CuZnSn	

**Table 3:** Transmission efficiency of each echo wave.

Times of Echo Wave of the Plate Bottom	Transmission Efficiency in Brazing Coupling	Transmission Efficiency in Dry Coupling
The first	144%	51.4%
The second	76.5%	28.6%
The third	47%	/
The fourth	28.6	/

**Table 4:** Measurement errors of thicknesses.

Standard Thicknesses	Measurement Errors by the Conventional Ultrasonic Transducer	Measurement Errors by the Waveguide bar System
6.210 mm	+0.67%	−1.50%
9.840 mm	+1.02%	+0.71%
20.104 mm	−0.49%	−0.40%

## Data Availability

No new data were created or analyzed in this study. Data sharing is not applicable to this article.
